# Structure and Dynamics of Drk-SH2 Domain and Its Site-Specific Interaction with Sev Receptor Tyrosine Kinase

**DOI:** 10.3390/ijms25126386

**Published:** 2024-06-09

**Authors:** Pooppadi Maxin Sayeesh, Mayumi Iguchi, Kohsuke Inomata, Teppei Ikeya, Yutaka Ito

**Affiliations:** Department of Chemistry, Tokyo Metropolitan University, 1-1 Minami-Osawa, Hachioji, Tokyo 192-0397, Japan; sayeesh@tmu.ac.jp (P.M.S.); iguchi-mayumi@ed.tmu.ac.jp (M.I.); kinomata@tmu.ac.jp (K.I.)

**Keywords:** GRB2, Drk, Src homology 2, signal transduction

## Abstract

The *Drosophila* downstream receptor kinase (Drk), a homologue of human GRB2, participates in the signal transduction from the extracellular to the intracellular environment. Drk receives signals through the interaction of its Src homology 2 (SH2) domain with the phosphorylated tyrosine residue in the receptor tyrosine kinases (RTKs). Here, we present the solution NMR structure of the SH2 domain of Drk (Drk-SH2), which was determined in the presence of a phosphotyrosine (pY)-containing peptide derived from a receptor tyrosine kinase, Sevenless (Sev). The solution structure of Drk-SH2 possess a common SH2 domain architecture, consisting of three β strands imposed between two α helices. Additionally, we interpret the site-specific interactions of the Drk-SH2 domain with the pY-containing peptide through NMR titration experiments. The dynamics of Drk-SH2 were also analysed through NMR-relaxation experiments as well as the molecular dynamic simulation. The docking simulations of the pY-containing peptide onto the protein surface of Drk-SH2 provided the orientation of the peptide, which showed a good agreement with the analysis of the SH2 domain of GRB2.

## 1. Introduction

The interaction between receptor tyrosine kinases (RTKs) and adaptor proteins characterises the initial phase of signal transduction in multicellular organisms; for facilitating the transmission of signals from the extracellular to the intracellular milieu [1]. Signalling initiates when the ligand-induced activation of RTKs leads to the autophosphorylation at the specific tyrosine residue sites. This process recruits adaptor proteins that harbour Src homology-2 (SH2) or phosphotyrosine (pY) binding domains towards the pY-containing region of RTKs. Since the SH2 domains play a central role in the recognition of the pY sites, disruption or over-activation of these adaptor proteins significantly impacts the generation of tumorigenesis [2,3]. Thus, considerable attention has been focused on investigating site-specific interactions between SH2 domains and RTKs. This is because of its critical importance in elucidating signalling pathways and its potential therapeutic applications [4].

SH2 is a modular domain motif comprising nearly 100 amino acids. Initially identified as a conserved sequence region between the oncoproteins Src and Fps, later on, they were subsequently discovered in numerous intracellular proteins [5]. Within the human genome, a total of 120 SH2 domains have been identified, which are present in 110 distinct proteins [6,7]. One of the best-known SH2-containing proteins in human is Growth Factor Receptor-Bound Protein 2 (GRB2), which plays a significant role in the Mitogen-Activated Protein Kinase (MAPK) pathway. GRB2 transmits growth factor signals downstream in response to RTKs, e.g., epidermal growth factor receptor (EGFR) activation [8], and establishes a link with Son of Sevenless homologue 1 (SOS1), a guanine nucleotide exchange factor of RAS proteins [9].

The *Drosophila* downstream of receptor kinase (Drk) is a homologue of GRB2, containing 211 amino acids [10], having a same domain structure as GRB2: two Src homology domains 3 (SH3) located at the N- and C-terminals (Drk-NSH3 and Drk-CSH3) and one SH2 domain (Drk-SH2) at the centre. Drk binds to activated RTKs, such as Sevenless (Sev), at the pY site (in case of Sev, Y2546) via the Drk-SH2 domain and the SH3 domains can bind with the proline-rich motifs (PRMs) in the disordered region of Sos of sevenless (Sos) and Daughter of sevenless (Dos) [11]. Drk is essential for the specification of photoreceptor R7 in the *Drosophila* eye. Additionally, Drk is present in various structures of the adult brain, including the antennal lobe (AL), ellipsoid body, and notably in the mushroom bodies (MBs). Further, its involvement has been identified in the development of olfactory learning and anaesthesia-resistant memory in *Drosophila* [12,13]. 

Solution NMR approach has been used to elucidate the structures of Drk-NSH3 [14] and Drk-CSH3 [15] domains in aqueous solution. The typical three-dimensional structure of SH3 domains consists of five β-strands. However, the Drk-NSH3 domain exhibits an equilibrium between the folded and unfolded states under the neutral condition, which is an exceptional structural behaviour compared to other SH3 domains [14]. A single mutation, T22G, stabilises the folded state of Drk-NSH3 [16]. Our structural analysis of Drk-CSH3 revealed that, unlike Drk-NSH3, Drk-CSH3 adopts only a folded conformation [15]. Drk interacts with PRMs such as the PxxPxR motif in Sos and the PxxxRxxKP motif in Dos [11,17]. Our previous reports of NMR titration experiments with peptides derived from Sos and Dos, along with the affinity comparison of both SH3 domains, revealed that both SH3 domains can bind with Sos and Dos with different affinities [18].

Although the structures of both SH3 domains and their interactions with Sos and Dos have been investigated, the complete interaction mechanism of Drk still remains unclear. This is primarily attributed to limited information regarding the structure and dynamics, especially those of Drk-SH2. In this study, we present the solution structure of the Drk-SH2 in the presence of the Sev-derived peptide containing a pY residue and characterise its recognition mechanism towards the RTK. Additionally, we explore the dynamics of the Drk-SH2 domain through relaxation experiments as well as molecular dynamics (MD) simulations. Finally, through docking simulations, we demonstrate the orientation of the pY-peptide on the surface of Drk-SH2.

## 2. Results

### 2.1. NMR Resonance Assignment and Structure Determination of Drk-SH2

The purified Drk-SH2 sample was found to be not sufficiently stable, and was suffered from the concentration-dependent aggregation, making long-term NMR measurements difficult. Noticeably, the solution structure of GRB2-SH2 domain complexed with the Shc-derived peptide (DDPSpYVNVQNLDK) has been reported [19]. For full-length GRB2, *K*_D_ against a pY-containing peptide, VPEpYINQSVPK, has been determined to be 0.713 ± 0.145 (μM) by isothermal titration calorimetry [20], and a similar *K*_D_ could be expected for the interaction of Drk-SH2 with pY-containing peptides. We therefore attempted measurements in the presence of a pY-containing peptide to overcome the concentration-dependent aggregation. The biochemical studies have reported that Drk-SH2 binds to the C-terminal tail of Sev at the tyrosine residue present in the position of 2546 (Y2546) [11]. This region possesses the common SH2-recognising motif, pYXNX, where X can be any amino acid. Consequently, the NMR experiments for the sequence-specific assignment of NMR resonances and the collection of NOE-derived distance restraints were performed in the presence of 1.5-fold higher amounts of a pY-containing peptide, KQLpYANEGVSR, corresponding to the resides, 2543–2553, in Sev. For further sample stabilisation, a non-detergent sulfobetaine, NDSB-195, was also added.

**Figure 1:**
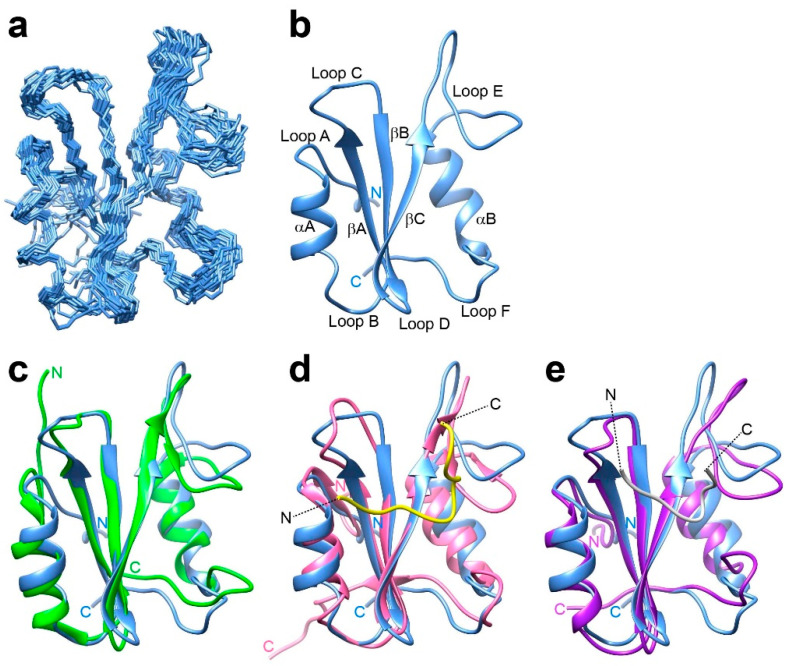
NMR solution structure of Drk-SH2 determined in the presence of the Sev-derived pY-containing peptide. (**a**) A superposition of the final 20 structures with the lowest CYANA target function values showing the backbone (N, C^α^, C′) atoms. (**b**) The lowest energy structure in the structure ensemble shown in (**a**). The positions of loop regions, α-helices and β-strands are indicated on the ribbon model. Comparisons of the Drk-SH2 structure (light blue) and the representative solution structure of GRB2-SH2 on its own (green, PDB ID: 6VK2) [23] (**c**), the solution structure of GRB2-SH2 in the complex with a Shc-derived peptide (DDP-SpYVNVQNLDK) (pink, PDB ID: 1QG1) [19] (**d**), and the crystal structure of GRB2-SH2 in the complex with a pY-containing peptide (KPFpYVNVEF) (magenta, PDB ID: 1BMB) [24] (**e**). In (**d**) and (**e**), the N- and C-termini of the pY peptides (shown in yellow (**d**) and in white (**e**)) are indicated.

The backbone (Supplementary Figure S1) and sidechain (Supplementary Figure S2) NMR resonance assignments of Drk-SH2 in the presence of the pY-peptide were achieved virtually completely by analysing various 3D triple-resonance NMR spectra. NOE-derived distance restraints were collected by analysing 3D ^15^N-separated and ^13^C-separated NOESY spectra. By incorporating NOE-derived distance restraints as well as backbone dihedral angle restraints, the 3D solution structure of Drk-SH2 was calculated with the program CYANA [21]. The energy minimisation of the resulting structure was achieved with the program AMBER 22 (Supplementary Table S1) [22]. The resulting 3D structure is well-converged, with a backbone RMSD of 0.85 Å to the mean coordinates. The superposition of the backbone heavy atoms of the final 20 structures with the lowest CYANA target function values is shown in Figure 1a. The obtained structure possesses an antiparallel β-sheet consisting of three strands (here we refer to them as them as βA, βB and βC) interposed between two α-helices (we refer to them as αA and αB), exhibiting a topology trend similar to the common “α + β” SH2-type domains. The positions of the β-strands and α-helices as well as the six loop regions (we refer to them as Loops A (N-terminal), B (between αA and βA), C (between βA and βB), D (between βB and βC), E (between βC and αB), and F (C-terminal) are indicated in Figure 1b.

Figure 1c–e show the structural comparisons of Drk-SH2 solution structure with the previously reported solution structures of the SH2 domain of GRB2 (GRB2-SH2); GRB2-SH2 on its own (green, PDB ID 6VK2) [23] and GRB2-SH2 in the complex with a Shc-derived pY-containing peptide, DDpYVNVQNLDK, (pink, PDB ID: 1QG1) [19], and the crystal structure of GRB2-SH2 in the complex with a pY-containing peptide, KPFpYVNVEF, (magenta, PDB ID: 1BMB) [24], respectively. The amino acid sequence comparison of both SH2 domains is presented in Supplementary Figure S3. GRB2-SH2 has a 68% sequence identity and an 83% sequence homology against Drk-SH2. The overall structure and the position of each secondary structure component were almost identical in all structures. On the other hand, the conformations of the regions corresponding to the Loops A, C, E and F in Drk-SH2 differ considerably from each other. However, these loop regions have also been reported to have different conformations between the crystal and solution structures of the pY peptide-bound state of GRB2-SH2 (Supplementary Figure S4) [23,24], which may be due to the common dynamic properties of these regions rather than differences between Drk and GRB2. 

### 2.2. Backbone Dynamics of Drk-SH2

**Figure 2:**
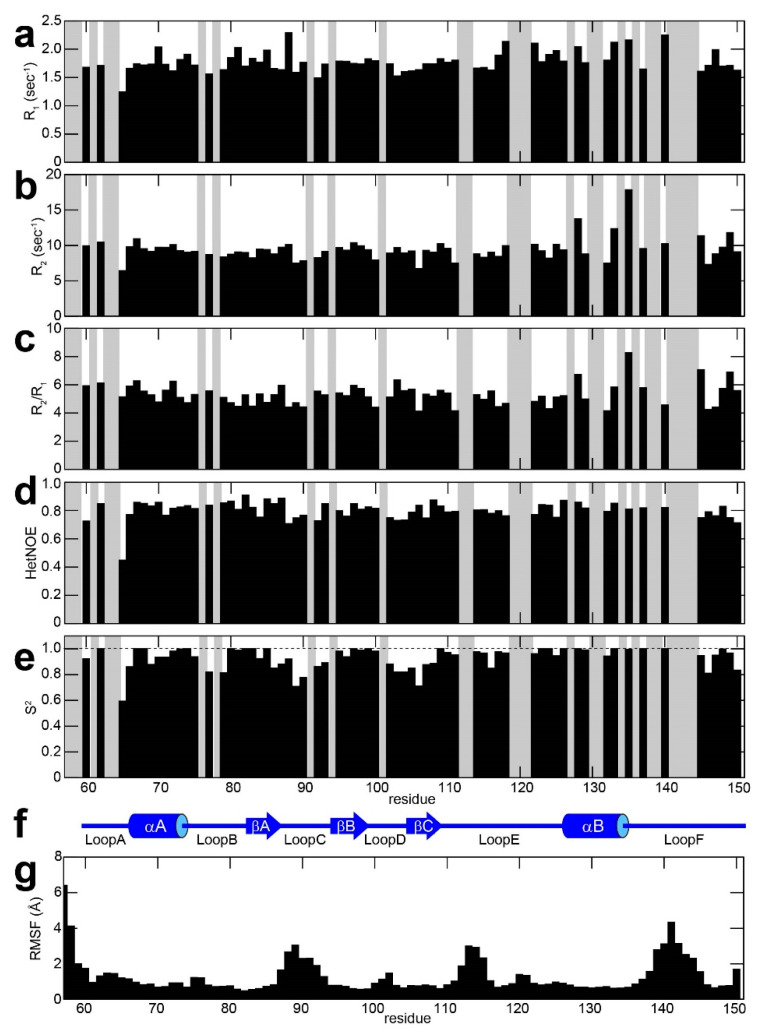
Plots of the backbone ^15^N R_1_ (**a**), R_2_ (**b**), R_2_/R_1_ (**c**), {^1^H}-^15^N NOE (**d**), and *S*^2^ values calculated by model-free analysis (**e**) for Drk-SH2 in the presence of the Sev-derived peptide, KQLpYANEGVSR, against the amino acid sequence. Grey bars correspond to proline residues and to residues for which ^1^H-^15^N correlation cross peaks were not analysed due to signal overlap. The secondary structure of Drk-SH2 is also shown (**f**). (**g**) The root-mean-square fluctuation (RMSF) plots against the amino acid sequence from the 2 µs molecular dynamic simulation of Drk-SH2.

To investigate the backbone dynamics of Drk-SH2, we measured the longitudinal (R_1_) and the transverse (R_2_) ^15^N relaxation rates as well as {^1^H}-^15^N NOEs for backbone amide ^15^N atoms in the presence of the pY-peptide. The plots of R_1_, R_2_, R_2_/R_1_, and {^1^H}-^15^N NOEs for Drk-SH2 against the amino acid residue numbers are shown in Figure 2a–d, respectively. The trend of R_2_/R_1_ values and {^1^H}-^15^N NOEs remained relatively constant even for most of the loop regions, which has a good agreement with the ^15^N-relaxation parameters reported for GRB2-SH2 in both the peptide-bound and the unbound states, where a mostly rigid profile was observed on the timescale for T_1_ and T_2_ relaxation [23]. Using these three relaxation data sets, we also performed model-free analysis with five model-free models assuming an isotropic global motion and evaluated the optimal model based on the Akaike Information Criterion (AIC). The model selection indicated that the relaxation data consist of global rotation with little intrinsic motions and do not contain the contribution of chemical exchange relaxation (corresponding to the *m*2 model in the relax software [25]). The obtained rotational correlation time was approximately 6.75 ns, which was in good agreement with that of GRB2-SH2 (6.5 ns) [23]. The values of obtained *S^2^* suggest that the Loop C region exhibits slightly larger local motion (Figure 2e). 

T_1_/T*_2_* NMR relaxation experiments elucidate the dynamics on the pico-to-nanosecond timescale, but do not clearly show the motion on nano-to-microsecond. In order to obtain deeper insights into the dynamics of the Drk-SH2 domain over longer timescales, we conducted a 2 μs molecular dynamic simulation (MD). The plot of the root-mean-square deviation (RMSD) is shown in Supplementary Figure S5, while the plot of the root-mean-square fluctuation (RMSF) is shown in Figure 2g. The RMSF plot revealed mainly three flexible loop regions: Loop C, E, and F. In the MD simulation of GRB2-SH2, a comparable pattern was also noted, in both the peptide-bound and unbound states, with particularly high RMSF values observed for the region corresponding to Loop C in Drk-SH2, which is estimated to be responsible for recognising pY [23]. The *S*^2^ values from the model-free analysis also showed larger dynamics in Loop C. Although the model selection in the model-free analysis with the relaxation data did not clearly validate the models considering explicit chemical exchange relaxation (*R*_ex_), combining these results of the MD simulation suggests that Loop C exhibits the dynamics on the pico-to-microsecond timescale. This relatively large motion of Loop C may contribute to the binding affinity of Drk-SH2 with the pY peptide. Loop E and F correspond the residues for which relaxation parameters were not obtained due to either no resonance assignments or a low peak intensity. The lack of the relaxation data for these loop regions may be due to the relatively larger dynamics on the microsecond timescale observed in the MD simulation. 

Hosoe et al. reported high RMSF values for the almost-similar loop region in the N-terminal SH2 domain of PI3K, which is a subunit of p85 showing ~25% sequence similarity with Drk-SH2 [26]. The reported MD data also indicated the formation of hydrogen bonds between the side-chain of S361, located in the loop region, with the pY present in the peptide derived from CD28 (SDpYMNMTPRRPG) [26]. Taken together with our results for Drk-SH2 and the results presented in these previously reported studies, the high RMSF commonly observed in this loop region may be directly related to the pY recognition mechanism; e.g., the capability to undergo additional structural changes to accommodate the pY residue.

### 2.3. Titration Experiment of Drk-SH2 with the Sev-Derived pY-Containing Peptide

As mentioned above, NMR experiments so far have been performed in the presence of an excess amount of the Sev-derived pY-peptide, KQLpYANEGVSR, due to the lack of long-term stability of Drk-SH2 on its own. To confirm that the pY peptide properly binds to the expected surface on Drk-SH2 and consequently contributes to the stability of the protein, stepwise titration experiments of ^15^N-labelled Drk-SH2 with the pY-peptide were performed. 

**Figure 3:**
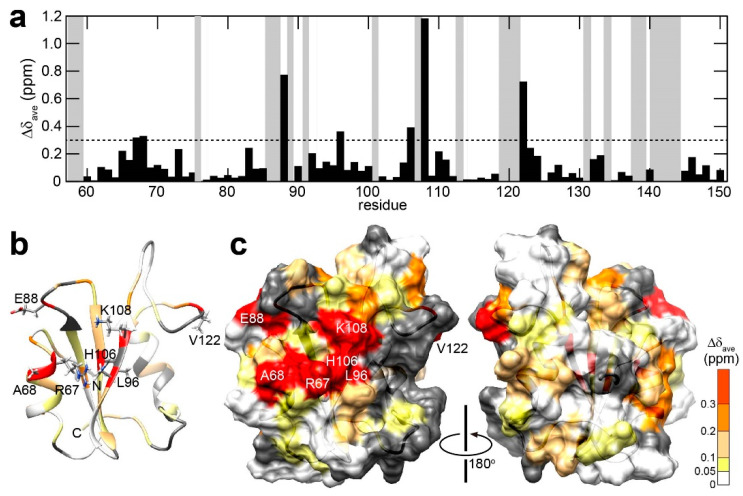
Chemical shift perturbation upon the titration of ^15^N-labelled Drk-SH2 with the Sev-derived pY-containing peptide (KQLpYANEGVSR). (**a**) The plot of chemical shift perturbation of backbone ^1^H^N^ and ^15^N nuclei. The mean shift difference Δδ_ave_ was calculated as [(Δδ^1^H^N^)^2^ + (Δδ^15^N/5)^2^]^1/2^ where Δδ^1^H^N^ and Δδ^15^N are the chemical shift differences (ppm) between Drk-SH2 on its own and in the presence of the peptide (Drk-SH2: peptide = 1:2). The proline residues as well as the residues for which ^1^H-^15^N correlation cross peaks were not assigned in at least one of the states are shown in grey. (**b**) Chemical shift perturbations are indicated on the solution structure of Drk-SH2. The residues which were affected in the presence of the peptide are coloured as follows: white (Δδ_ave_ < 0.05 ppm); yellow (0.05 ppm ≤ Δδ_ave_ < 0.1 ppm); pale orange (0.1 ppm ≤ Δδ_ave_ < 0.2 ppm); orange (0.2 ppm ≤ Δδ_ave_ < 0.3 ppm); red (Δδ_ave_ ≥ 0.3 ppm). The residues which showed significant chemical shift perturbations (Δδ_ave_ ≥ 0.3 ppm) are annotated and the side-chain atoms for these residues are shown. (**c**) The distribution of the perturbed residues is also exhibited on the surface of Drk-SH2. Significant perturbations are found on the localised surface of the protein.

The backbone ^1^H/^15^N resonance assignment of Drk-SH2 on its own was obtained by analysing 3D HNCO and HNCA spectra measured in the absence of the pY peptide, with reference to the assignments obtained for the peptide-bound state (Supplementary Figure S6). The overlays of 2D ^1^H-^15^N HSQC spectra of Drk-SH2 from the titration experiments are presented in Supplementary Figure S7, which revealed that the pY-peptide interaction with Drk-SH2 undergoes in the slow exchange regime on the NMR time scale. As mentioned above, the *K*_D_ for the interaction of full-length GRB2 with VPEpYINQSVPK has been determined to be 0.713 ± 0.145 (μM) [20]. The slow exchange regime found in the titration experiments suggests that the *K*_D_ for the interaction of Drk-SH2 with the Sev-derived pY-containing peptide is likely to be in the similar range as was obtained for full-length GRB2. The plot of the chemical shift differences between the peptide-unbound and bound states are shown against the amino acid sequence in Figure 3a. The chemical shift differences are also indicated on the solution structure of Drk-SH2 (Figure 3b,c), indicating that the chemical shift changes caused by the peptide binding was observed over a wide area of the molecule.

Major chemical shift differences were predominantly localised in the region encompassing αA, βA, βB, and βC, which comprise the previously identified interaction pocket with the pY peptide for GRB2-SH2 [19,24,27,28]. Specifically, residues A68, E88, L96, H106, and K108 exhibited substantial chemical shift differences, likely due to proximity to the aromatic ring of pY, resulting in a ring current effect. This suggests that Drk-SH2 recognises the pY residue in a similar manner to GRB2-SH2. In the pY peptide utilised in the GRB2-SH2 complex (DDpYVNVQNLDK), which also belongs to the pYXNX motif [19], the side-chain of asparagine at the second position from pY (pY + 2) forms a pair of hydrogen bonds with the backbone amide and carbonyl groups of K109 in GRB2-SH2, thus stabilising its interaction. The largest chemical shift perturbation was observed for K108 of Drk-SH2, corresponding to K109 of GRB2-SH2, which precludes confirmation of a comparable interaction in Drk-SH2. Meanwhile, the reason why ^1^H-^15^N correlation cross peaks could not be assigned for F107 may be due to chemical exchange between multiple conformations in the region in the absence of the pY-containing peptide. The high mobility of this region is also inferred from the results of the MD simulation.

Interestingly, significant chemical shift changes exceeding 0.3 ppm were also observed in V122, despite their substantial distance from the pY-binding pocket. Typically, pY peptides adopt U-shaped structures upon binding to SH2 domains [28], in which both N- and C-termini extend away from the protein surface, centred around the pY residue. Given this conformation, it is unlikely that these distant residues directly interact with the peptide. In particular, V122, which is markedly distant from the pocket, suggests that its chemical shift change is not due to the peptide extending to these regions. This raises the possibility that either the pY peptide does not bind with Drk-SH2 in the U-shaped structure, as it does with GRB2-SH2, or that the peptide binding induces a conformational change in the area surrounding to V122. Relatively large chemical shift differences were also observed in the αB helix region, which is also distant from the pY-binding pocket and proximal to V122. CPMG relaxation experiments of GRB2-SH2 suggested that, while the domain possesses two independent subdomains and exhibits conformational exchange within its domain in the absence of the EGFR-derived pY peptide, these dynamics are clearly suppressed in the presence of the peptide [23]. Considering the motional changes of GRB2-SH2, the large chemical shift differences may indicate that the Sev-derived pY peptide binding caused structural changes in the region of αB helix and V122.

### 2.4. The Docking Simulations of pY-Peptide on Drk-SH2

It has been reported that some SH2 domains showed binding preference towards the specific residues at the pY + 2, pY + 3 and pY + 4 positions in the peptide ligand [1]. In order to visualise the orientation of the Sev-derived pY-containing peptide on the surface of Drk-SH2 and compare them with the experimental data, docking simulations were performed using the HADDOCK 2.4 [29,30]. The calculation was performed using the regions with significant chemical shift perturbations as “active” residues in HADDOCK (see Section 4). V112 was excluded from the category of the active residue despite exhibiting substantial chemical shift perturbations, due to its location being quite far from the estimated binding pocket and the absence of chemical shift perturbation data for the region connecting the pocket and V112.

**Figure 4:**
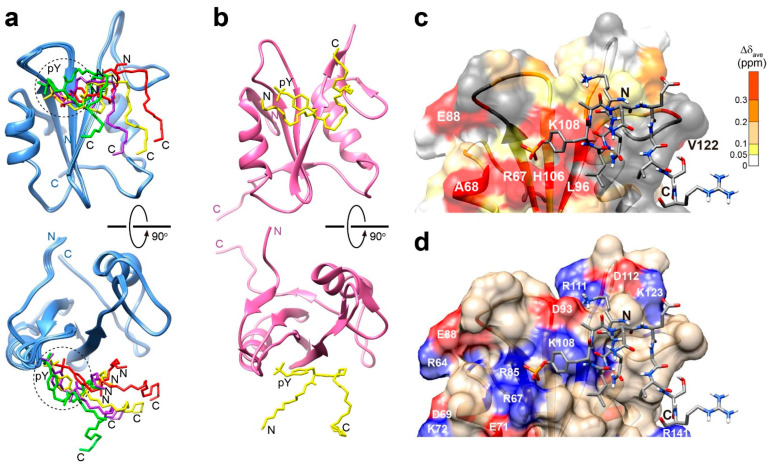
The models of the interactions of Drk-SH2 with the pY-containing peptide (KQLpYANEGVSR) derived from the docking simulations. (**a**) The top four structures with lowest energy are shown, in which the backbone heavy atoms of the peptide as well as the side-chains of the phosphotyrosine residues are exhibited. The N- and C-termini of the peptides are also indicated. (**b**) The solution structure of GRB2-SH2 in the complex with another pY-containing peptide (DDpYVNVQNLDK) (PDB ID: 1QG1)) is shown for comparison. (**c**) Chemical shift perturbations upon the titration of ^15^N-labelled Drk-SH2 with the Sev-derived pY-containing peptide (KQLpYANEGVSR) are indicated on the representative (with the lowest energy) model structure. The residues showing significant chemical shift changes are colour-coded and annotated as in Figure 3b,c. (**d**) Negatively and positively charged residues on the surface of Drk-SH2 are annotated and colour-coded red and blue, respectively, on the representative model structure. In (**c**,**d**), the N- and C-termini of the pY-containing peptide are indicated.

According to the docking results (Figure 4a), the peptide was oriented on the surface of Drk-SH2 in a distorted U-shaped manner. The overall U-shape conformation of the peptide resembled the interaction mode which has been reported previously for pY peptides with SH2 domains [19,24,28]. The solution structure of GRB2-SH2 complexed with the peptide (DDpYVNVQNLDK, PDB ID 1QG1) is shown in Figure 4b for comparison. The pY residue was positioned close to a shallow cleft formed between the αA helix and Loop C. This cleft comprises primarily positively charged residues, R67, R85, (H106), and K108. R67 and H106 are the residues that exhibited large chemical shifts in the titration experiments (Figure 4c,d). In the docking simulations, the guanidium moiety of R67 and R85 showed more proximity towards the pY (Figure 4d).

Although the orientation of pY within the cleft between the αA helix and the Loop C was similar between the Drk-SH2 and GRB2-SH2 complexes, notable differences were found for a couple of residues following the pY (pY+2-4) relative to the protein surface. In the GRB2-SH2 complex, these residues demonstrated closer contact with the protein surface, whereas in the Drk-SH2 complex, the N-terminal region of the pY-containing peptide had more proximity, unlike the C-terminus (pY+2-4). These inconsistencies may be attributed to the amino acid variations at two X positions in the pYXNX motif and the presence of a lysine residue in the N-terminus of the Sev-derived peptide (KQLpYANEGVSR). As described above, the X positions in the motif are occupied by alanine and glutamate in the Sev-derived peptide, in contrast to two valines for the peptide in the GRB2 complex, which may contribute the different interaction mode of the Drk-SH2 complex from the rigid hydrogen bonds in the GRB2 complex. Meanwhile, the N-terminus of the Sev-derived peptide was positioned toward D93 in the Loop C (Figure 4d), whereas the corresponding region in the GRB2 complex was located away from the protein surface (Figure 4b), probably due to two aspartates influencing interactions with the positive-charge patches.

## 3. Discussion

The interactions between RTKs and adaptor proteins play significant roles in various signal transduction pathways, thus contributing a wide range of cellular processes including cell proliferation, apoptosis, etc. In *Drosophila*, around 20 RTKs have been identified, and some of them have been explored for specific functions. Drk plays a pivotal role in the exploration of cellular activities [31], as it binds to the RTKs via its SH2 domain and interacts with Sos and Dos through its other domains, NSH3 and CSH3. The biophysical properties (3D structures, dynamics, etc.) of the SH3 domains in Drk have been investigated by solution NMR spectroscopy [14,15], and their binding activities to proline-rich motifs have also been studied [14,15,18]. Structural biology studies of SH2 domains have so far only been performed on the human homologue GRB2 [19,23,24]. This study now provides detailed knowledge of Drk-SH2.

In this study, we explored the solution NMR structure of Drk-SH2 and noted its similarity to the common SH2 domain conformation. Comparing it with GRB2-SH2, we observed slight conformational changes in the loop regions, including the loop C region, which corresponds to the pY-binding surface. Analysis of backbone ^15^N-relaxation parameters for Drk-SH2 revealed a rigid profile in the presence of the pY-containing peptide. However, the MD simulation of Drk-SH2 on its own with a microsecond time-scale showed a different flexibility profile, notably with large RMSF values in five loop regions. Excluding the N- and C-terminal regions, the Loop C region exhibited the most significantly higher RMSF values, suggesting potential conformational changes to accommodate pY. The recognition mechanism of pY may likely occur in the following manner: initially, the activation of RTKs leads to dimerisation and phosphorylation of tyrosine, which attracts the SH2 domain of Drk. The pY is recognised by electrostatic interaction between the phosphate group of pY and the side-chains of the residues R67, R88, and H106, thus altering the orientation to the Loop C region.

Drk consists of three domains, yet a full-length structure for Drk has not been reported to date. The predicted structure by AlphaFold2 [32] for the full-length Drk (UniProt Accession ID: Q6YKA8) indicates that contacts between SH2 and NSH3 domains are closer than those between the SH2 and CSH3 domains. The present study successfully provided structural bases for the interaction between Drk-SH2 and activated Sev. However, the analysis did not consider the influence of inter-domain interactions. Although the SH2 domain is the primary pY reader, the interaction between the SH2 domain and the other SH3 domains may also contribute pY recognition. An analysis of the full-length Drk could further provide insights into Drk-regulated molecular events in *Drosophila.*

## 4. Materials and Methods

### 4.1. Purification of Drk-SH2

The gene encoding Drk-SH2 (residues 60–150) was cloned into a pET-14b (Novagen, Madison, WI, USA) vector, and the resulting plasmid was introduced into the *Escherichia* coli BL21-CodonPlus(DE3)-RIPL (Agilent, Santa Clara, CA, USA) strain.

For NMR experiments, uniformly ^13^C and ^15^N-labelled (^13^C/^15^N-) Drk-SH2 was prepared as an N-terminal GST-tagged protein with a TEV cleavage site, by growing the transformed bacteria at 37 °C in M9 minimal medium containing 1 g/L ^15^NH_4_Cl (Cambridge Isotope Laboratories, Inc.) and 2 g/L [^13^C_6_] D-glucose (Cambridge Isotope Laboratories, Inc., Tewksbury, MA, USA) as the sole nitrogen and carbon sources, respectively. At an OD_600nm_ of~0.5, protein expression was induced by the addition of isopropyl thio-β-D-thiogalactoside to a final concentration of 1 mM. After 16 h of further growth at 20 °C, the cells were harvested. All the purification procedures described below were carried out at 4 °C. The harvested cell pellet was suspended in the lysis buffer [50 mM Tris-HCl (pH 7.5), 1 mM DTT, 1 mM EDTA and 200 mM NaCl], and were disrupted by sonication for 30 min on ice in the presence of hen egg lysozyme (0.1 mg/mL). The cell debris was clarified by centrifugation at 60,000 *g* for 1 h. The GST-tagged Drk-SH2 was purified using a Glutathione S-transferase affinity column (Cytiva) pre-equilibrated with the lysis buffer. The Drk-SH2-containing fractions eluted with 20 mM Glutathione were collected, and the GST cleavage was performed overnight by uTEV3 protease (Addgene) at room temperature. Note that three glycine residues from the TEV-cleavage site and a GG linker remained at the N-terminus (G57, G58 and G59) after the cleavage. Further purification of Drk-SH2 was achieved using a Superdex 75 pg HiloadTM 16/600 (Cytiva) gel filtration column pre-equilibrated with purification buffer [50 mM Tris-HCl (pH 7.5), 1 mM DTT, 1 mM EDTA and 200 mM NaCl]. The final Drk-SH2 fractions were concentrated (300 μM) and dissolved in the NMR buffer [20 mM KH_2_PO_4_/K_2_HPO_4_ (pH 7.4), 200 mM KCl, 1 mM DTT, 0.5 M NDSB-195 (Merck) and 0.05% NaN_3_] including 10% D_2_O for NMR lock using an Amicon Ultra-15 centrifugal concentrator with a 3kDa molecular-size cut off (Merck).

### 4.2. NMR Spectroscopy

All NMR experiments were performed at 25 °C either on a Bruker Avance-III HD 600 MHz spectrometer equipped with a cryogenic triple-resonance probehead or a Bruker Avance-III HD 500 MHz spectrometer equipped with a room-temperature triple-resonance probehead. All 2D NMR spectra were processed using the Azara 2.8 software suite (Wayne Boucher and Department of Biochemistry, The University of Cambridge). For all 3D NMR data, a non-uniform sampling scheme was used for the indirectly observed dimensions to reduce experimental time. In brief, approximately 1/8 of the points were selected in a pseudo-random fashion from the conventional regularly spaced grid of t1, t2 points. Two-dimensional quantitative maximum entropy (QME) processing [33] was used for the two indirectly acquired dimensions after processing the directly acquired dimension (t3) by Fourier transformation using the Azara 2.8 software. All spectra were visualised and analysed using the CcpNmr Analysis 2.5.1 software [34].

Backbone and side-chain NMR resonances of Drk-SH2 were assigned by analysing 3D triple-resonance NMR spectra, HNCA, HN(CO)CA, HN(CA)CO, HNCO, CBCANH, CBCA(CO)NH, HBHA(CBCACO)NH, H(CCCO)NH, CC(CO)NH, and HCCH-TOCSY, measured on uniformly ^13^C/^15^N-labelled Drk-SH2 (300 μM) in the presence of 1.5 times excess amount of the peptide containing a phosphotyrosine residue, KQLpYANEGVSR, derived from Sev (Eurofins Genomics, Louisville, KY, USA). During the assignment process the result from a FLYA automated analysis [35] were used as a reference. Three-dimensional ^15^N-separated and ^13^C-separated NOESY-HSQC spectra with a mixing time of 100 ms were measured on the same sample, and analysed for the collection of NOE-derived distance restraints. 

Backbone NMR resonances of Drk-SH2 on its own (150 µM) were assigned by analysing 3D HNCA and HNCO, which were measured in the absence of the pY-containing peptide.

The longitudinal (R_1_) and the transverse (R_2_) relaxation rates of backbone ^15^N nuclei were measured at 25 °C using uniformly ^15^N-labelled Drk-SH2 (300 μM) in the presence of KQLpYANEGVSR peptide (450 μM). Each experiment was acquired in a pseudo-3D manner. A total of 8 relaxation delays in the range of 15–1005 ms and 6 delays between 14 and 110 ms were used for the R_1_ and R_2_ experiments, respectively. The hetronuclear NOE ratios were obtained from intensities in experiments recorded with (2 s relaxation delay followed by 3 s saturation) and without (relaxation delay of 5 s) saturation. Calculation of relaxation parameters and model-free analysis were performed using the relax software 5.0 [25]. Assuming that Drk-SH2 is nearly spherical with isotropic rotational diffusion, five model-free models can be selected in relax: m0 = {}, m1 = {*S*^2^}, m2 = {*S*^2^, *τ*_e_}, m3 = {*S*^2^, *R*_ex_}, m4 = {*S*^2^, *τ*_e_, *R*_ex_}. The variables in the parentheses for each model correspond to order parameters (*S*^2^), effective correlation times (*τ*_e_), and chemical exchange rates (*R*_ex_), which are individually calculated for each main chain N^H^ to represent local motion. All the models include the rotational correlation times. The model selection procedure was determined using the Akaike Information Criterion (AIC).

NMR titration experiments of Drk-SH2 with the KQLpYANEGVSR peptide were performed by measuring a series of 2D ^1^H-^15^N HSQC spectra on ^15^N-labelled Drk-SH2 (160 μM) in the presence of the peptide with various concentrations. Specifically, the experiments were carried out at 25 °C with stepwise increase of the peptide concentrations (1:0, 1:0.1, 1:0.2, 1:0.3, 1:0.4, 1:0.5, 1:0.7, 1:1, 1:1.25, 1:1.5, 1:1.75, and 1:2).

### 4.3. Structure Calculation of Drk-SH2

Drk-SH2 structures were calculated with the program CYANA [21] version 3.9.9 using automated NOE assignment and torsion angle dynamics, which was started from 100 conformers with random torsion angle values. The CYANA simulated annealing schedule was applied with 50,000 torsion angle dynamics steps. Backbone torsion angle restraints obtained from chemical shifts by utilising TALOS-N server [36] were added to the input for CYANA. The 20 conformers with the lowest final CYANA target function values were refined by using Amber version 22 with ff19SB force field and OPC water model in the presence of the experimental restraints. 

### 4.4. Molecular Dynamics Simulations

The resulting NMR structure of Drk-SH2 was used for the molecular dynamics (MD) simulation. MDs were performed using the Amber version 22 using the ff19SB force field and TIP3P parameters for the protein and water molecules. Three chlorides were added to the system to maintain the electric neutrality. The resulting solvated molecule was energy-minimised. Analysis of simulations was conducted using VMD software 1.9.4.

### 4.5. Molecular Docking Simulations

The docking calculations were performed using the HADDOCK 2.4 (high-ambiguity driven protein–protein docking) webserver (https://wenmr.science.uu.nl/haddock2.4, accessed on 30 April 2024) following standard protocols [29,30]. Briefly, the structures of Drk-SH2 and the respective pY-peptide were uploaded to the HADDOCK webserver, selecting “protein or protein-ligand” and “peptide” as the “kind of molecule”, respectively. The active residues for docking, including Drk-SH2 residues with large chemical shift perturbations (T66, R67, A68, L83, I84, E88, Q105, H106, K108, L110, and R111) along with the three peptide residues pY, pY+1, and pY+2, were specified as input parameters for HADDOCK. All other docking parameters were kept at their default settings.

## Data Availability

NMR assignment data have been deposited to the BioMagResBank (BMRB) with an accession number 36655. The final atomic coordinates of the structures of Drk-SH2 have been deposited in the Protein Data Bank with the accession code 8Z0N.

## Supplementary Materials

The following supporting information can be downloaded at: https://www.mdpi.com/article/10.3390/ijms25126386/s1.
