# Supplementary material for: Structure and Dynamics of Drk-SH2 Domain and Its Site-Specific Interaction with Sev Receptor Tyrosine Kinase
Source: Int J Mol Sci. 2024 Jun 9;25(12):6386. doi: 10.3390/ijms25126386 (PMC11203457; doi:10.3390/ijms25126386)
Supplement: Supplementary file 1 [file ijms-25-06386-s001.zip › ijms-3013384-supplementary.pdf]

Supplementary data for:

Structure and Dynamics of Drk-SH2 Domain and Its Site-Specific Interaction with Sev Receptor Tyrosine Kinase

Pooppadi Maxin Sayeesh[1], Mayumi Iguchi[1], Kohsuke Inomata[1], Teppei Ikeya[1*] and Yutaka Ito[1*]

[1]*Department of Chemistry, Tokyo Metropolitan University, 1-1 Minami-Osawa, Hachioji, Tokyo, 192-0397, Japan*

*Corresponding authors: ito-yutaka@tmu.ac.jp (Y. Ito) and tikeya@tmu.ac.jp (T. Ikeya)

Table of Contents

Supplementary Figure S1. The 2D ^{1}H-^{15}N-HSQC spectrum of Drk-SH2 in the presence of a Sev-derived phosphotyrosine-containing peptide.
Supplementary Figure S2. Schematic drawing summarising the backbone and sidechain assignments of Drk-SH2.
Supplementary Table S1. NMR structure statistics for Drk-SH2.
Supplementary Figure S3. Comparison of amino acid-sequences around SH2 domains in Drk and GRB2.
Supplementary Figure S4. Comparisons of the solution and crystal structures of GRB2-SH2 in the complex with phosphotyrosine-containing peptides.
Supplementary Figure S5. The root mean square deviations (RMSD) plots against the elapsed time from the 2 μs molecular dynamic simulation of Drk-SH2.
Supplementary Figure S6. The 2D ^{1}H-^{15}N-HSQC spectrum of the Drk-SH2 domain in the absence of Sev-derived pY-containing peptide.
Supplementary Figure S7. Overlays of 2D ^{1}H-^{15}N HSQC spectra from multipoint titrations of ^{15}N-labelled Drk-SH2 with the Sev-derived pY-containing peptide (KQLpYANEGVSR).

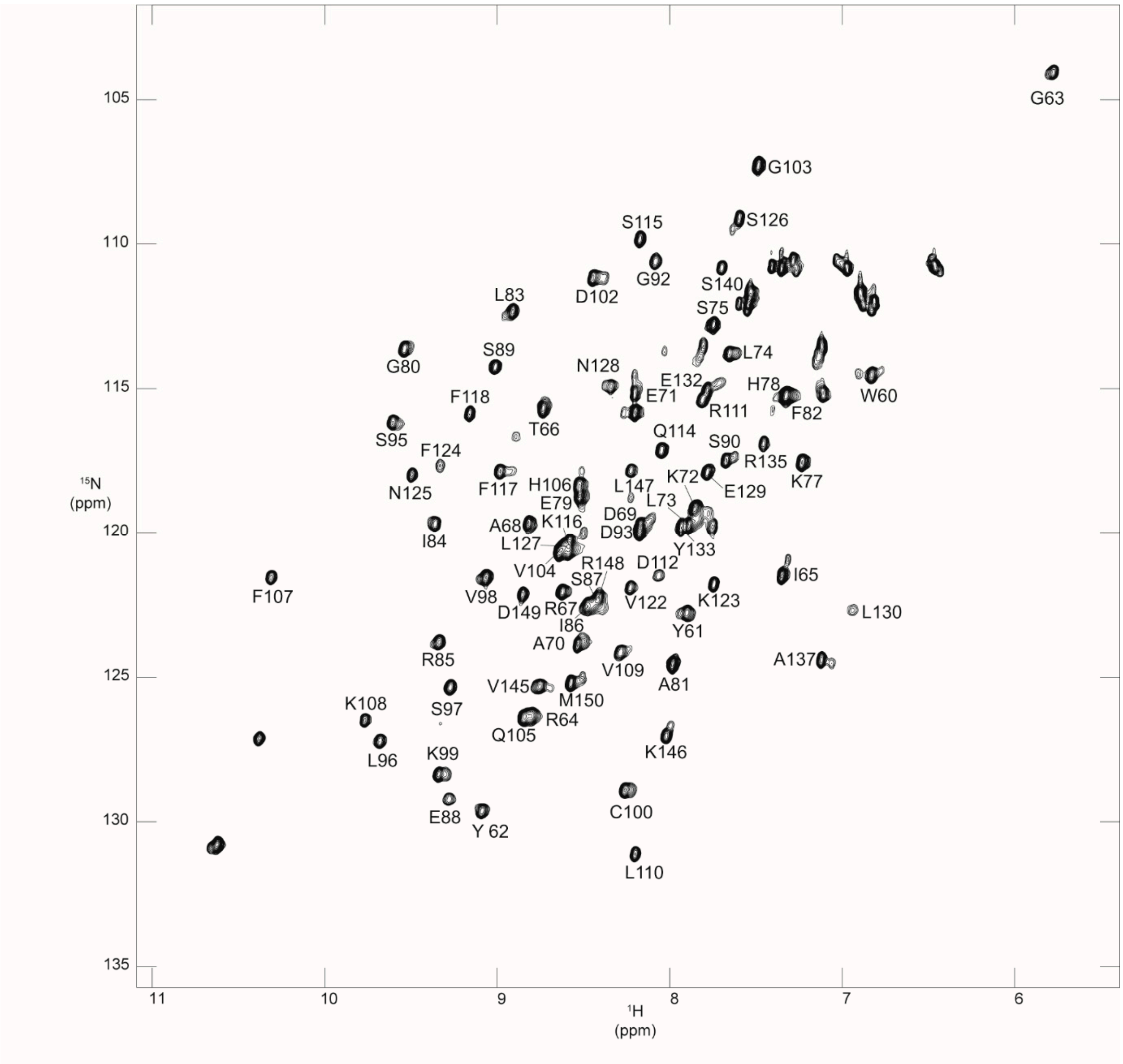

Supplementary Figure S1. The 2D ^{1}H-^{15}N-HSQC spectrum of the SH2 domain of Drk (Drk-SH2) in the presence of a Sev-derived phosphotyrosine (pY)-containing peptide (KQLpYANEGVSR). Cross peaks due to backbone amide groups are labelled with their corresponding assignments.

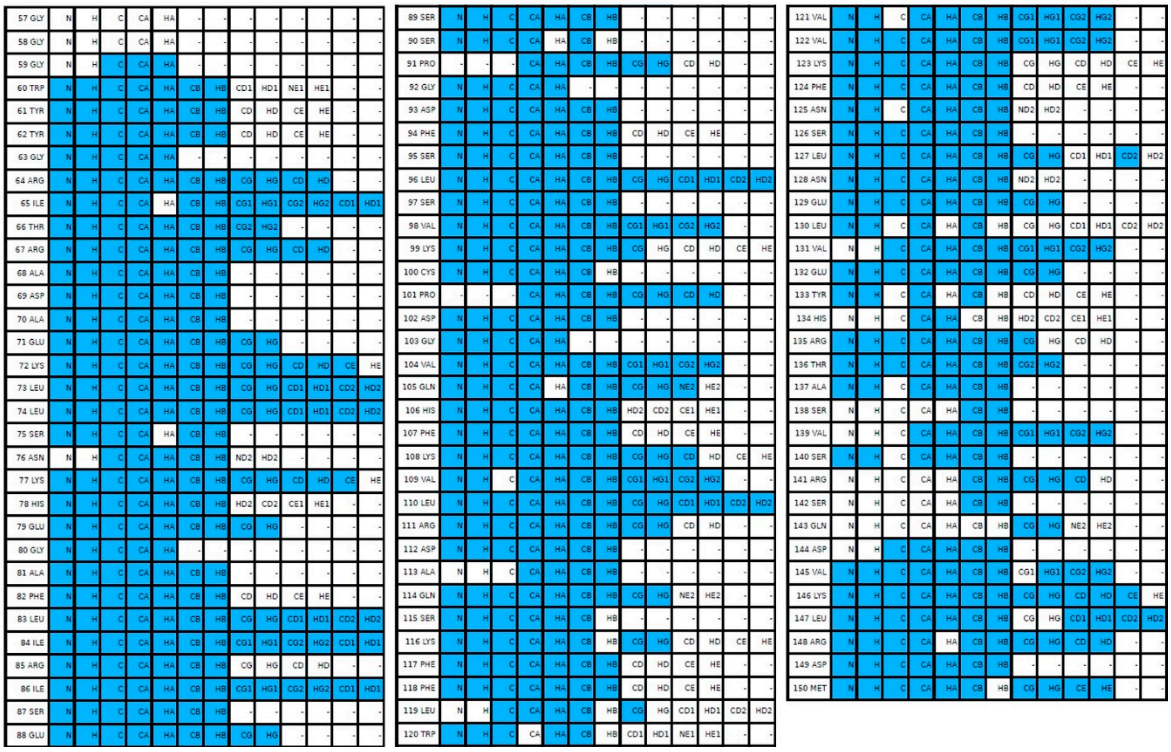

Supplementary Figure S2. Schematic drawing summarising the backbone and side-chain resonance assignments of Drk-SH2. Blue and white columns indicate assigned and unassigned atoms, respectively.

Supplementary Table S1. NMR structure statistics for Drk-SH2

Quantity[a]	Final structures
Assigned $^{1}H/^{13}C/^{15}N$ chemical shifts	392/333/79
NOE distance restraints[b]	
Short	465
Medium	137
Long-range	320
Dihedral angle restraints (ϕ/ψ)	156
Max. distance restraint violation (Å)	0.15 ± 0.03
Max. dihedral angle restraint violation (º)	4.89 ± 0.80
Deviations from idealized geometry:	
Bond lengths (Å)	0.0149 ± 0.0001
Bond angles (º)	1.86 ± 0.03
AMBER energy (kcal/mol)	– 18,508 ± 1,507
AMBER vdW energy (kcal/mol	– 1,899 ± 103
Ramachandran plot statistics[c] (%)	
Most favoured	90.2
Additionally allowed	9.8
Generously allowed	0.0
Disallowed	0.0
Number of distance violations[d]	10
Number of dihedral violations[d]	2
Backbone RMSD (Å)[e]	0.85 ± 0.21
All heavy atom RMSD (Å)	1.53 ± 0.22

[a]Where applicable, the average value and the standard deviation over the 20 energy-refined conformers obtained by the program Amber 22 with the ff19SB force field and OPC water model in the presence of the experimental restraints. CYANA calculations were started from 100 conformers with random torsion angle values, simulated annealing with 50,000 torsion angle dynamics steps was applied.
[b]Short/medium/long-range distance restraints derived from NOESY spectra.
[c]Percentage of residues in the most favoured/additionally allowed/generously allowed/disallowed regions of the Ramachandran plot according to the program PROCHECK.
[d]Number of violations found in at least 15 of the 20 final conformations.
[e]The backbone RMSD was calculated for $^{13}C^{\alpha}$, $^{13}C'$ and ^{15}N atoms.

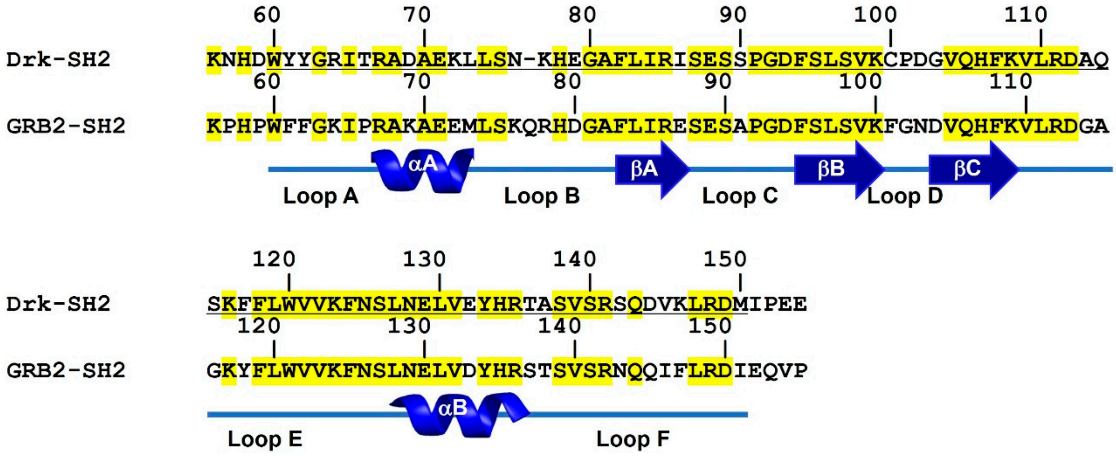

Supplementary Figure S3. Comparison of amino acid-sequences around SH2 domains in Drk and GRB2, in which residues conserved in both sequences are highlighted in yellow. Residues corresponding to the Drk-SH2 used in this study are underlined. The position of each secondary structure component is also shown.

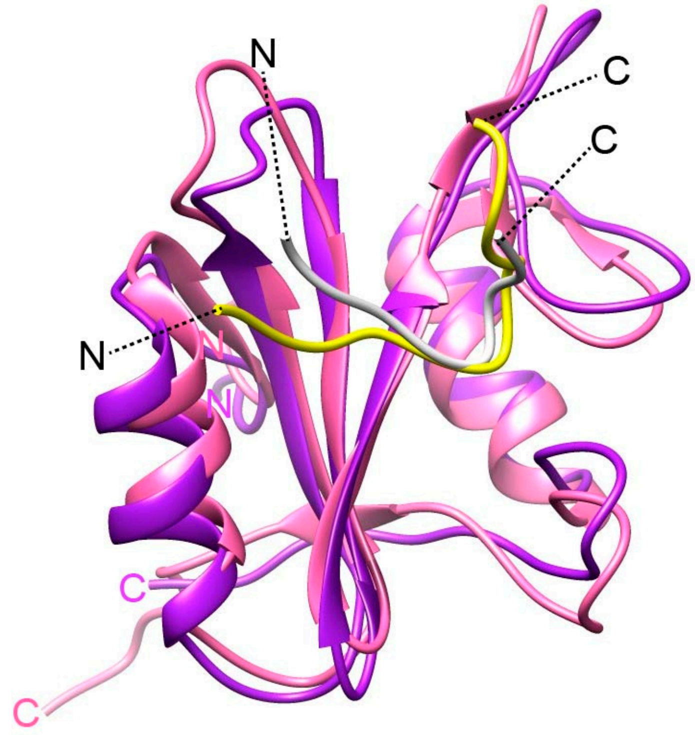

Supplementary Figure S4. Comparisons of the solution structure of GRB2-SH2 in the complex with a Shc-derived peptide (DDPSpYVNVQNLDK) (pink, PDB ID: 1QG1) and the the crystal structure of GRB2-SH2 in the complex with a pY-containing peptide (KPFpYVNVEF) (magenta, PDB ID: 1BMB). The N- and C-termini of the pY peptides (shown in yellow for 1QG1 and in white for 1BMB) are indicated.

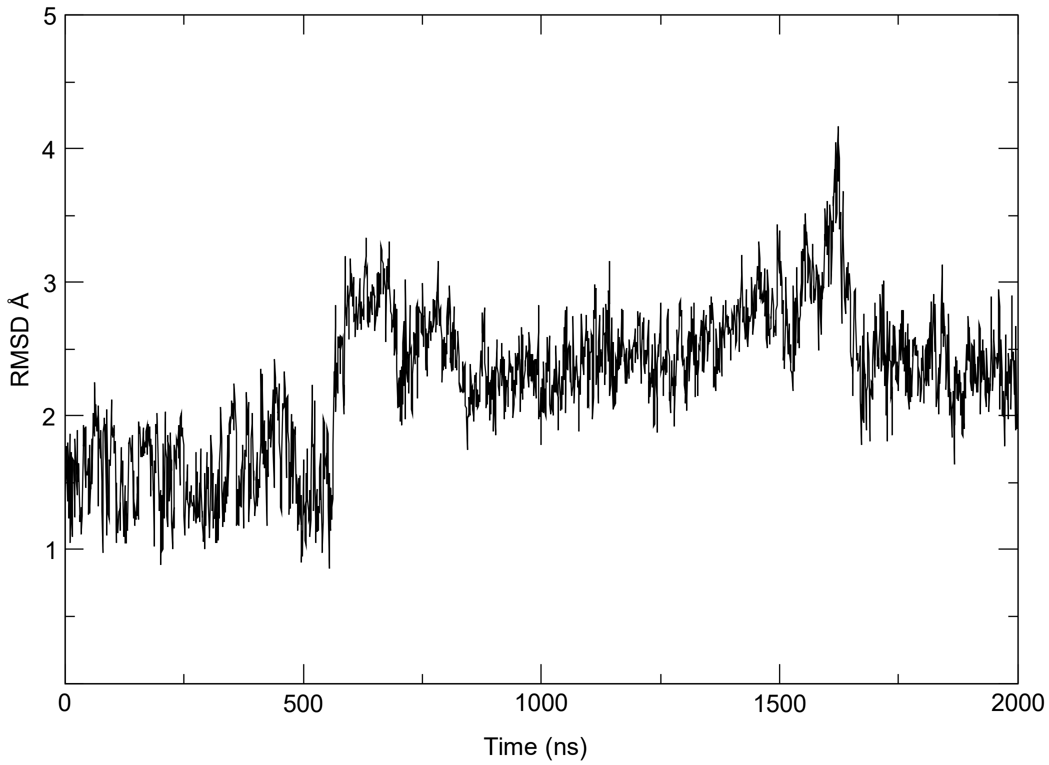

Supplementary Figure S5. The root mean square deviations (RMSD) plots against the elapsed time from the 2 µs molecular dynamic simulation of Drk-SH2.

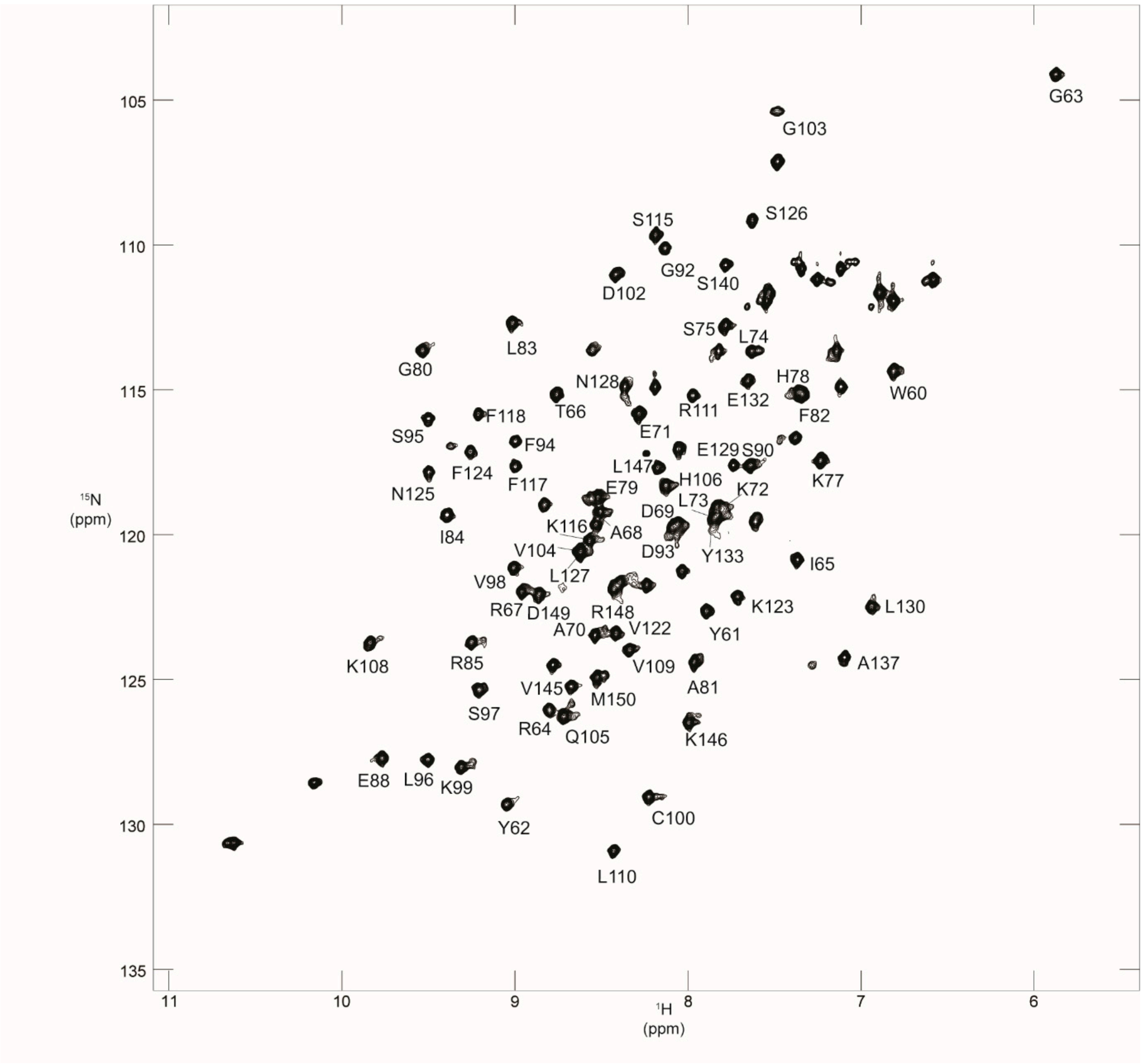

Supplementary Figure S6. The 2D ^{1}H-^{15}N-HSQC spectrum of the Drk-SH2 domain in the absence of Sev-derived pY-containing peptide. Cross peaks due to backbone amide groups are labelled with their corresponding assignments.

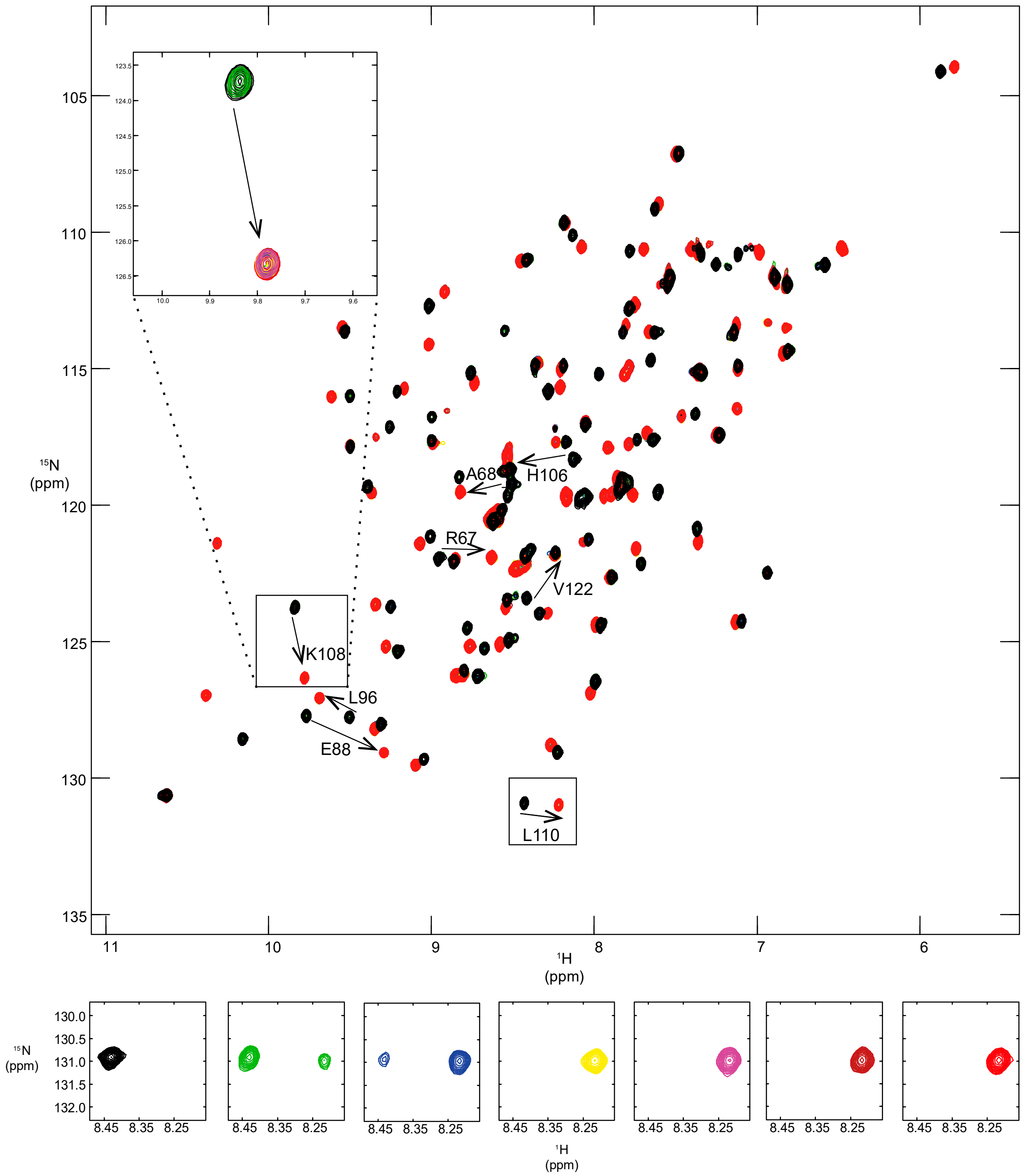

Supplementary Figure S7. Overlays of 2D ^{1}H-^{15}N HSQC spectra from multipoint titrations of ^{15}N-labelled Drk-SH2 with the Sev-derived pY-containing peptide (KQLpYANEGVSR). Cross peaks showing significant chemical shift changes are labelled with their corresponding assignments. For cross peaks corresponding to K108 and L110, enlarged spectra are also shown. In this figure, the colour codes of ^{1}H-^{15}N correlation cross-peaks at each titration point, showing the molar ratio of Drk-SH2:pY-peptide, are as follows: black (1:0); green (1:0.1); blue (1:0.2); yellow (1:0.3); magenta (1:0.5); brown (1:1); red (1:2)
